# Supplementary material for: Allosteric activation of an ion channel triggered by modification of mechanosensitive nano-pockets
Source: Nat Commun. 2019 Oct 10;10:4619. doi: 10.1038/s41467-019-12591-x (PMC6787021; doi:10.1038/s41467-019-12591-x)
Supplement: Supplementary file 1 — Supplementary Information [file 41467_2019_12591_MOESM1_ESM.pdf]

Supplementary Information

Allosteric activation of an ion channel triggered by modification of mechanosensitive nano-pockets

Charalampos Kapsalis[1], Bolin Wang[1,2,3], Hassane El Mkami[4,5], Samantha J. Pitt[6], Jason R. Schnell[7], Terry K. Smith[8], Jonathan D. Lippiat[3], Bela E. Bode[5,8] and Christos Pliotas[1,2,3,5]

[1]*Biomedical Sciences Research Complex, School of Biology, University of St Andrews, KY16 9ST, UK*
[2]*Astbury Centre for Structural Molecular Biology, University of Leeds, LS2 9JT, UK*
[3]*School of Biomedical Sciences, Faculty of Biological Sciences, University of Leeds, LS2 9JT, UK*
[4]*School of Physics and Astronomy, University of St Andrews, KY16 9SS, UK*
[5]*Centre of Magnetic Resonance, University of St Andrews, KY16 9ST, UK*
[6]*School of Medicine, University of St Andrews,KY16 9TF, UK*
[7]*Department of Biochemistry, University of Oxford, OX1 3QU, UK*
[8]*Biomedical Sciences Research Complex, School of Chemistry, University of St Andrews, KY16 9ST, UK*

Correspondence and requests for materials should be addressed to Christos Pliotas (email: c.pliotas@leeds.ac.uk)

Supplementary Figures

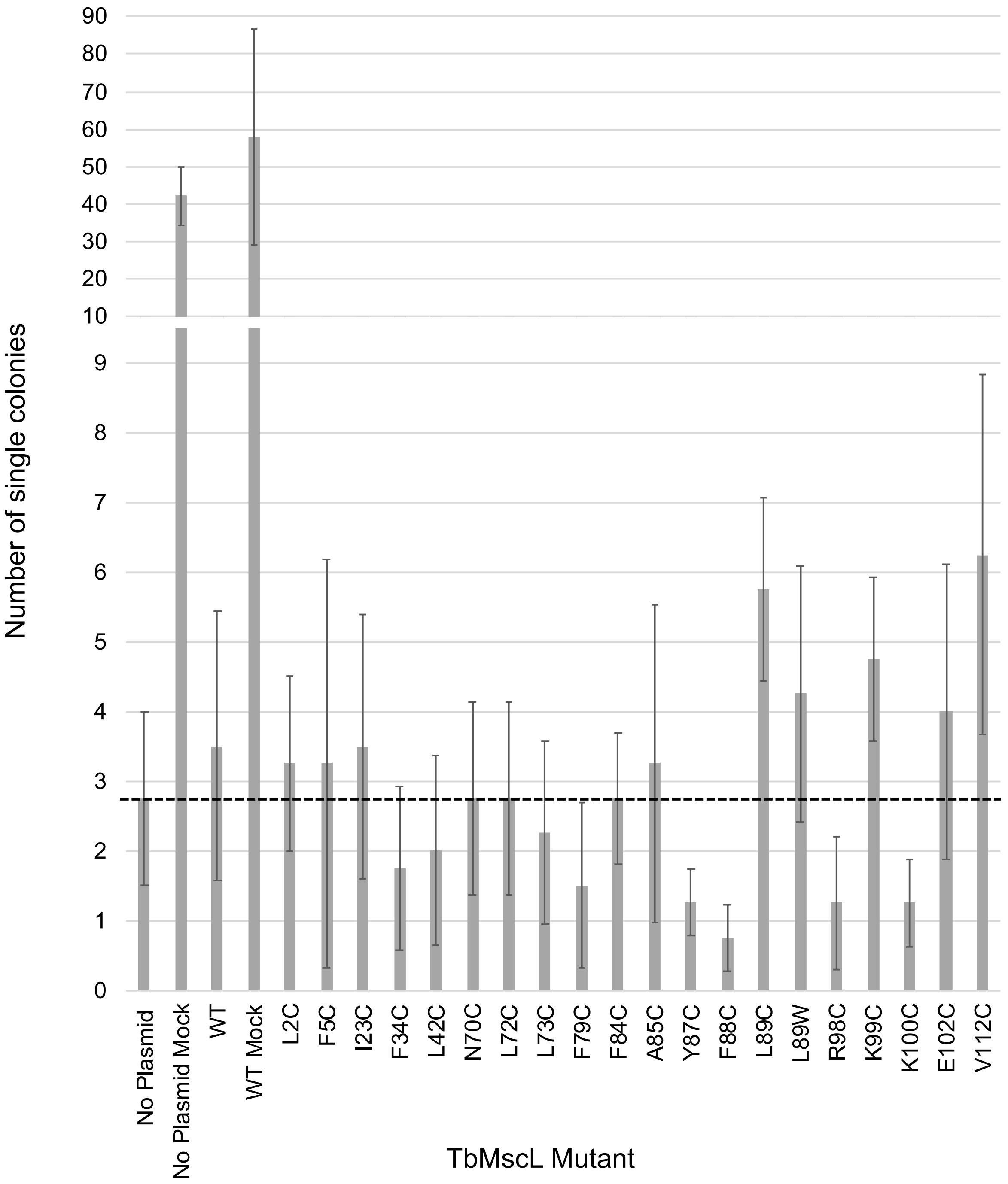

Supplementary Figure 1: Osmotic downshock (cell viability) assays of TbMscL mutants. Assays were independently repeated in quadruplicates. The majority single overexpressed *Tb*MscL mutants, provided variable, ranging from low to minimal (or no) protection to *E. coli* MJF612 cells (ΔmscL, ΔmscS, ΔmscK and ΔybdG) when subjected to a "mild" (0.3 M NaCl) hypoosmotic shock, similar to *Tb*MscL WT protein. The latter does not offer any protection against "harsh" osmotic shock (0.5 M NaCl), unlike *Ec*MscL WT protein, accounting for large differences between the two organisms (i.e. *Mtb* vs *Ec*). The dashed line represents the threshold level of protection. Errors are based on the standard deviation of independently performed experiments (quadruplicates).

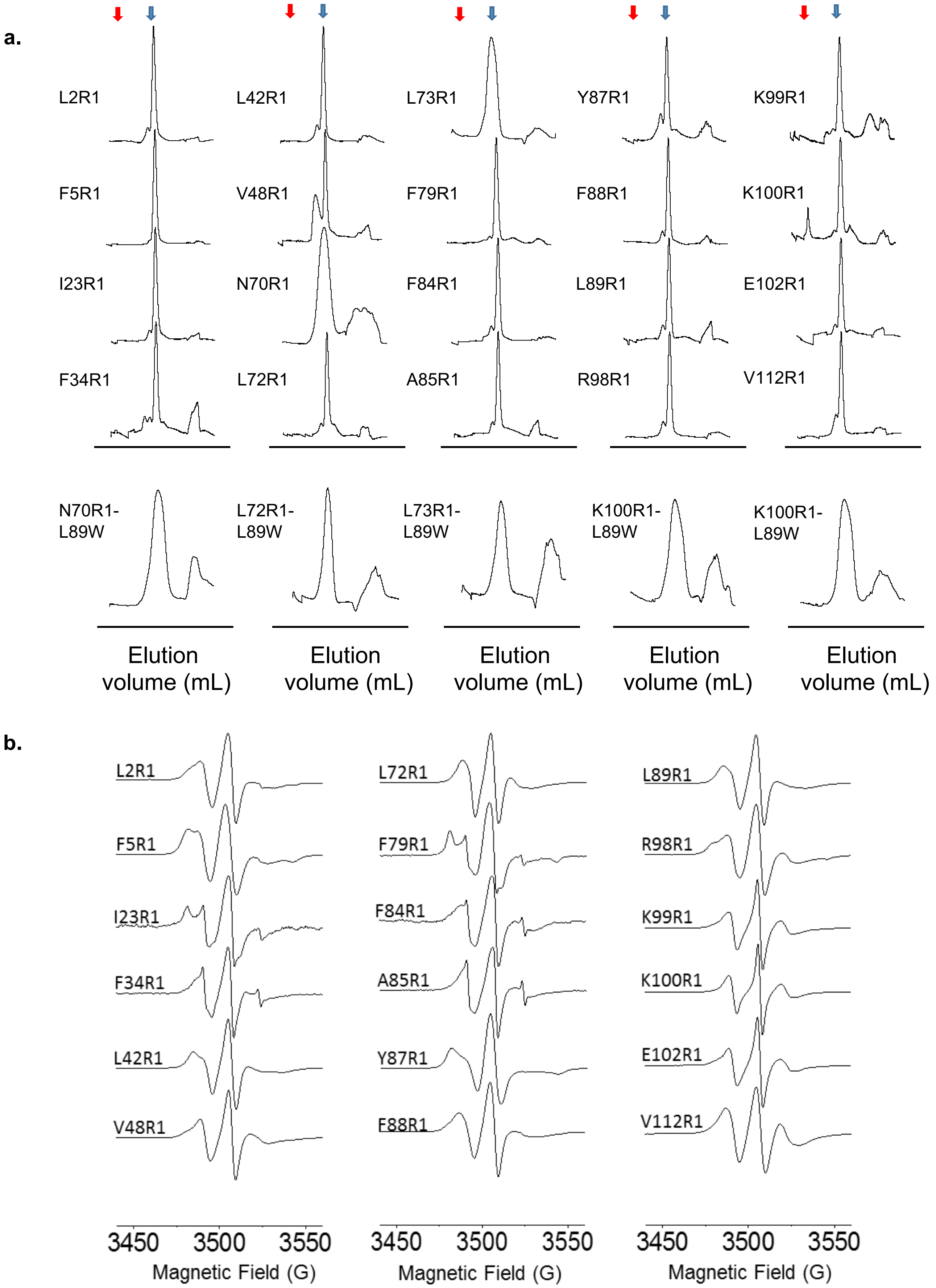

Supplementary Figure 2: Homogeneity and spin labelling assessment of PELDOR modified mutant samples. Size exclusion chromatography profiles of TbMscL mutants. Blue arrows denote the expected elution volume for the pentameric channel and red arrows show the retention volume of the column used in each case. A Superdex200 16/60 column was used in most cases, apart from N70R1, L73R1 and all double mutants for which a Superdex 10/30 column was used instead. **b.** CW-EPR spectra of single cysteine MTSSL-modified TbMscL mutants.

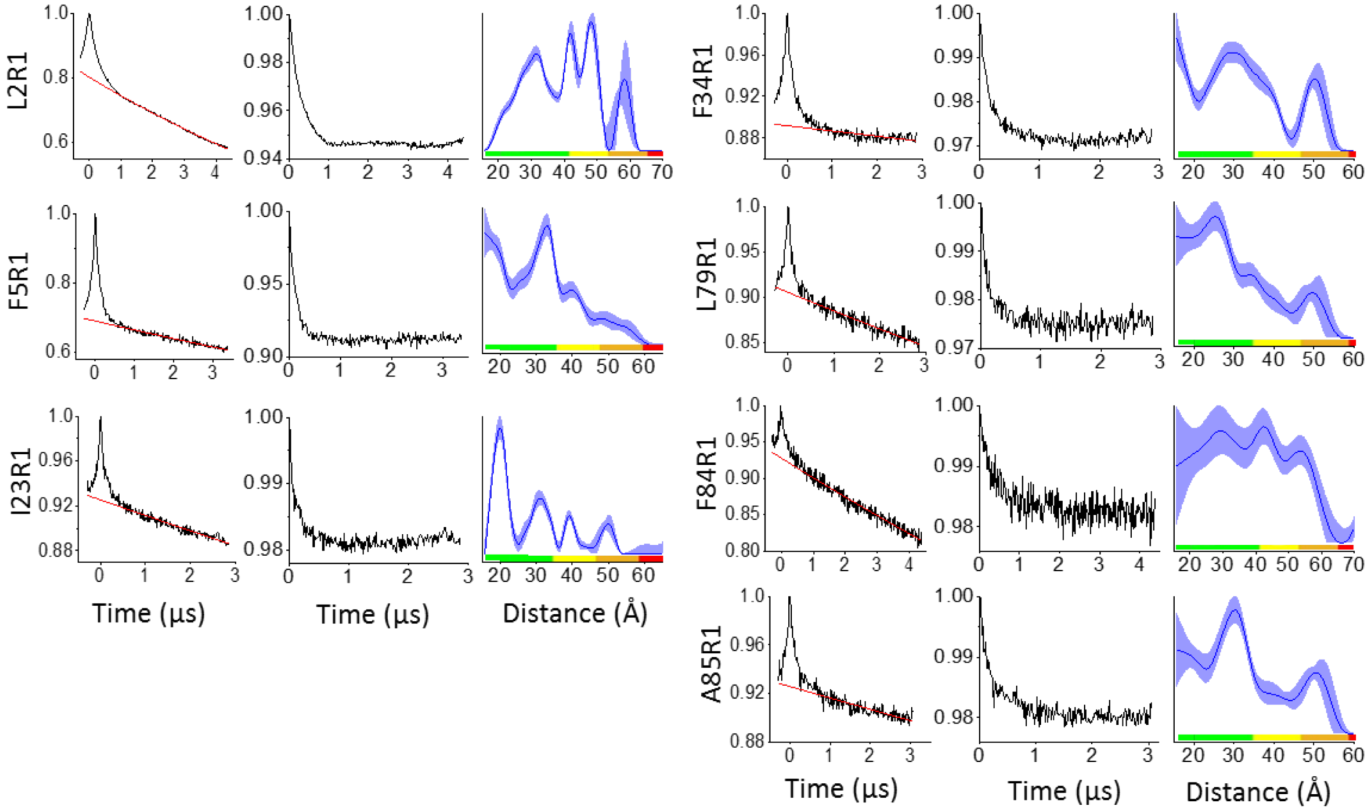

Supplementary Figure 3: Excluded from further analysis mutants. Raw background uncorrected (left column) and corrected (middle column) PELDOR time domain traces and resulting distance distributions (right column) of TbMscL mutants which presented <20% labelling efficiency and were excluded from further analysis.

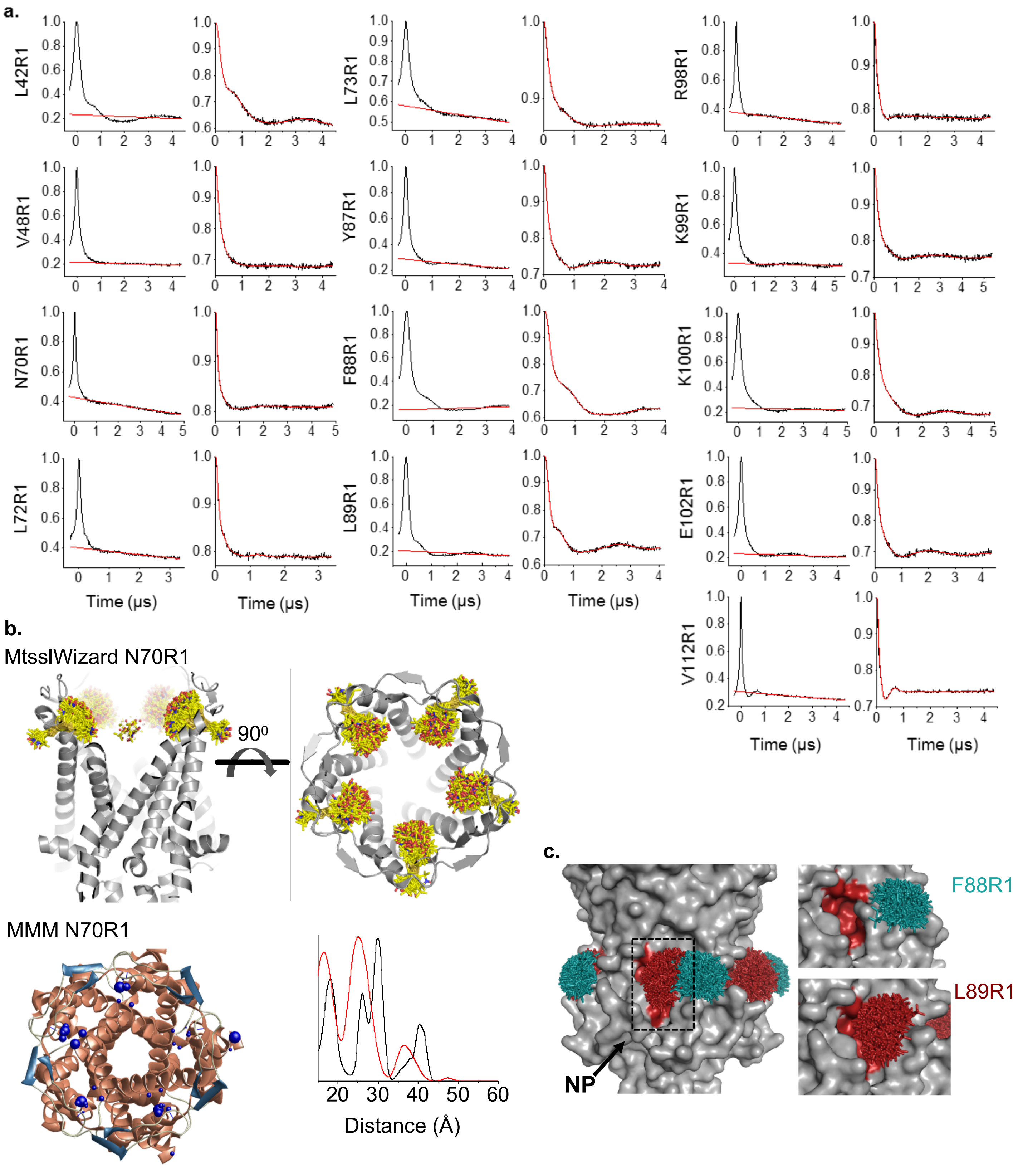

Supplementary Figure 4: Spin label modelling and NPs a. PELDOR time domain spectra before background correction (left, red line) and after (right, fitting function in red), of TbMscL mutants in solution. The background functions and resulting fitting correspond to the best fit calculated by the "Validation" tool in DeerAnalysis. Positive background slope in the case of F88R1 was retained as it arises from an incomplete oscillation. This would only be overcome by time window extension, which was not possible due to reduced signal intensity, or by truncating the time domain spectra prior to fitting, which would compromise our measurements. **b.** MTSSL rotamers of N70R1 as predicted from *in silico* spin labelling using MtsslWizard (yellow sticks, top) and MMM (blue spheres, bottom left). Resulting distance distributions MtsslWizard (red line) and MMM (black line). **c.** R1 rotamers of L89 (red sticks) blocking the entrance of TbMscL's NP (red surface). R1 rotamers of adjacent F88 residue (green sticks) do not restrict NP access , having no effect on MscL's state (Fig 2a).

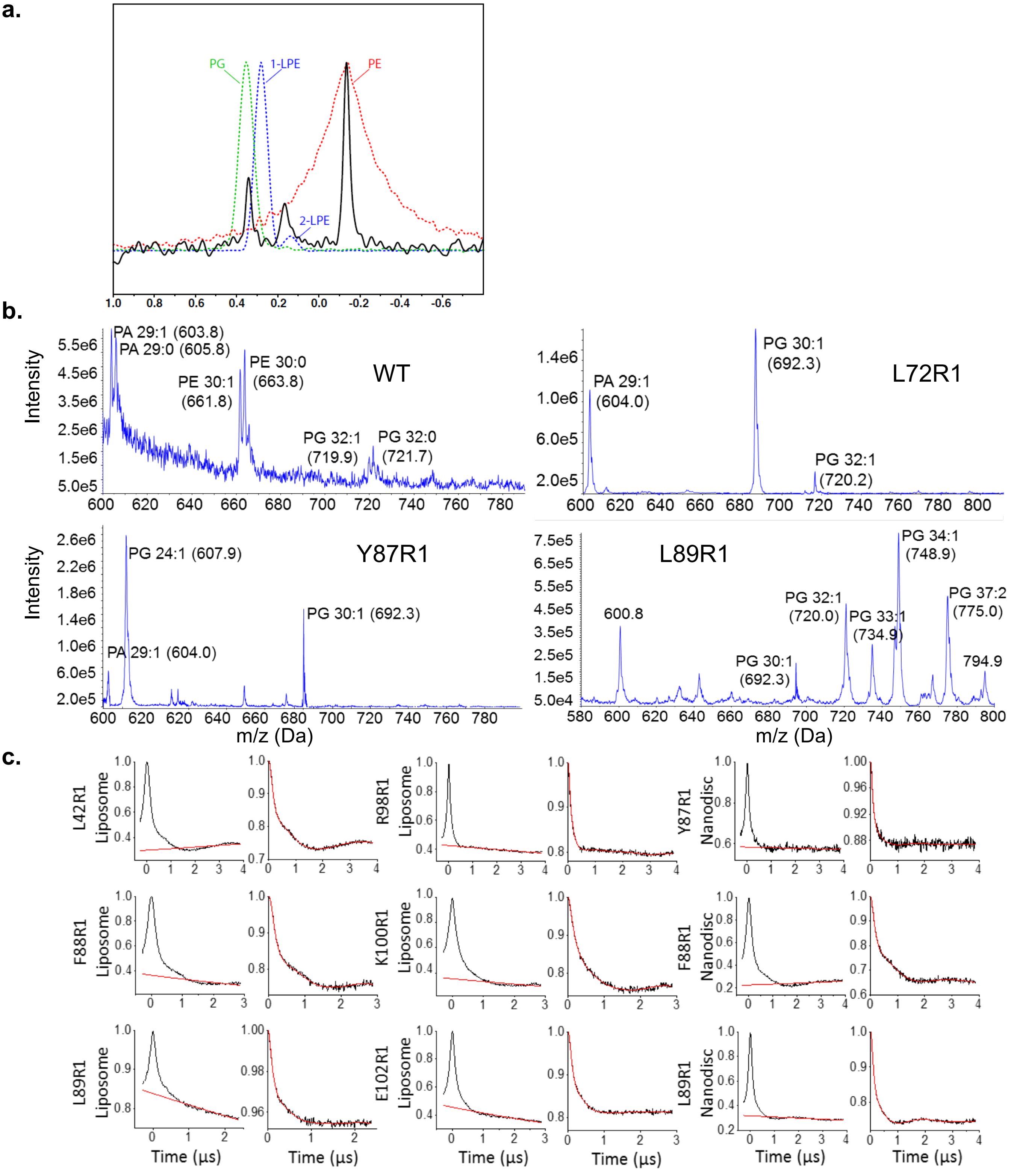

Supplementary Figure 5: Endogenous lipid analysis of purified TbMscL protein samples and gating transitions in lipids. a. Detection of endogenous lipids of WT TbMscL detergent purified sample (solution) (black trace) by ^{31}P-NMR spectroscopy. 1- and 2-*lyso*-phosphatidyl ethanolamine (1-/2-LPE, blue trace), phosphatidyl–ethanolamine (PE, red trace) and –glycerol (PG, green trace) standards are also presented for comparison. The peak intensity ratio between endogenous PE (black trace, red arrow) and PG (black trace, green arrow) lipids is ~3, similar to PE to PG ratio found in *E. coli* membranes **b.** Representative ES-MS of extracted endogenous lipids from purified TbMscL modified mutants. **c.** PELDOR time domain spectra before background correction (left, background shown in red) and after (right, fitting function shown in red), of TbMscL reconstituted in lipid bilayers. The background functions and resulting fitting correspond to the best fit calculated by the "Validation tool" of DeerAnalysis. Positive background slopes in liposome L42R1 and nanodisc F88R1 samples were retained as they arise from incomplete oscillations. This would only be overcome by time window extension using the validation tool, which was not possible due to reduced signal intensity, or by truncating the time domain spectra prior to fitting, which would compromise our measurements. However, for any fitting procedure we followed no significant change in distance distribution mean distance, width and shape was observed.

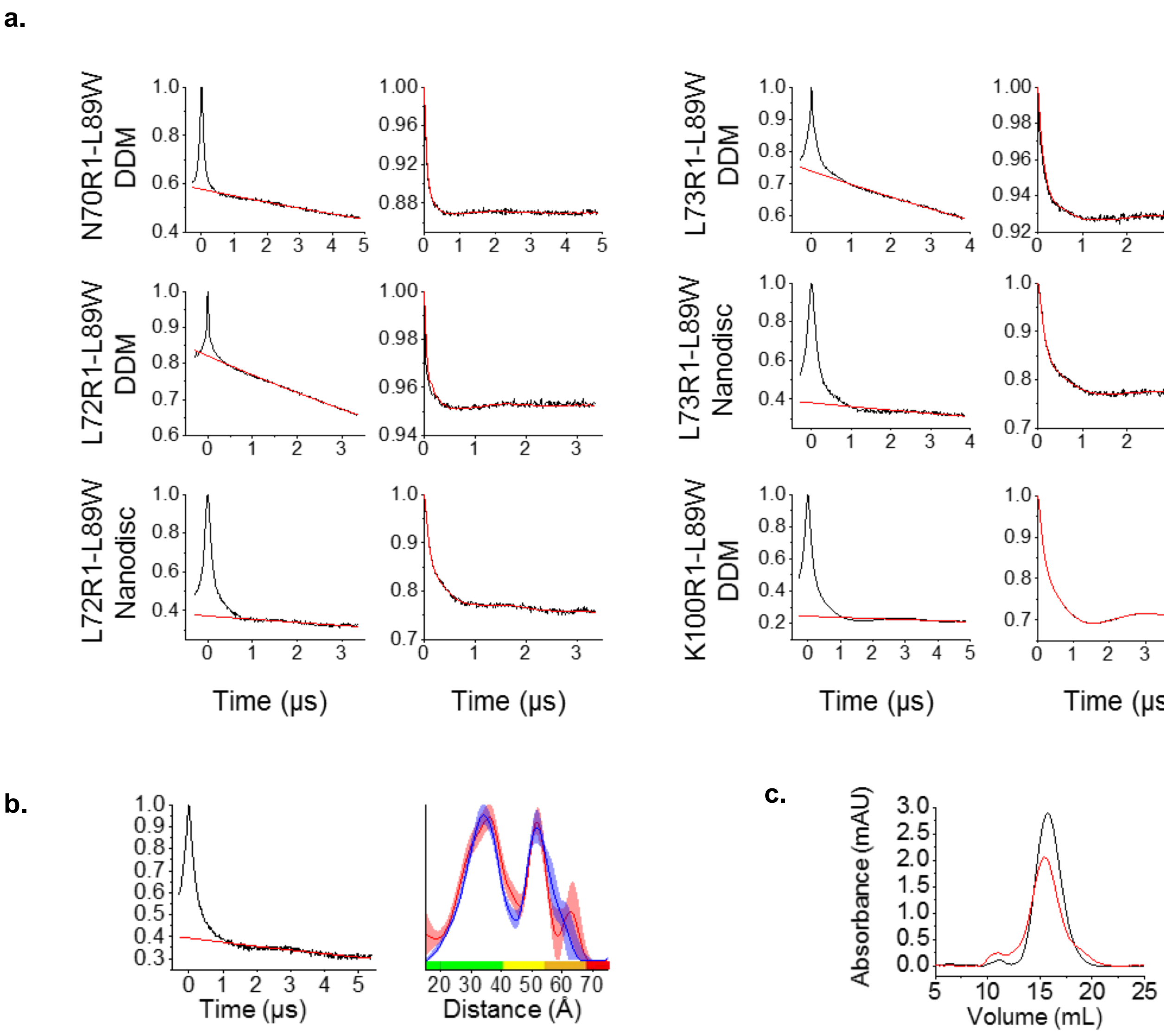

Supplementary Figure 6: Folding and stability assessment of modified mutants after PELDOR a. PELDOR time domain traces before background correction (left, background shown in red) and after (right, fitting function shown in red), of TbMscL L89W-paired EPR-reporter mutants N70R1, L72R1, L73R1 and K100R1 in DDM solution and nanodiscs**. b.** K100R1-F88W time domain trace (left) and distance distribution (right, red) compared to that of single-cysteine K100R1 (right, blue). **c.** SEC profiles of thawed DDM samples E102R1 (black) and L72R1-L89W (red) after PELDOR measurements.

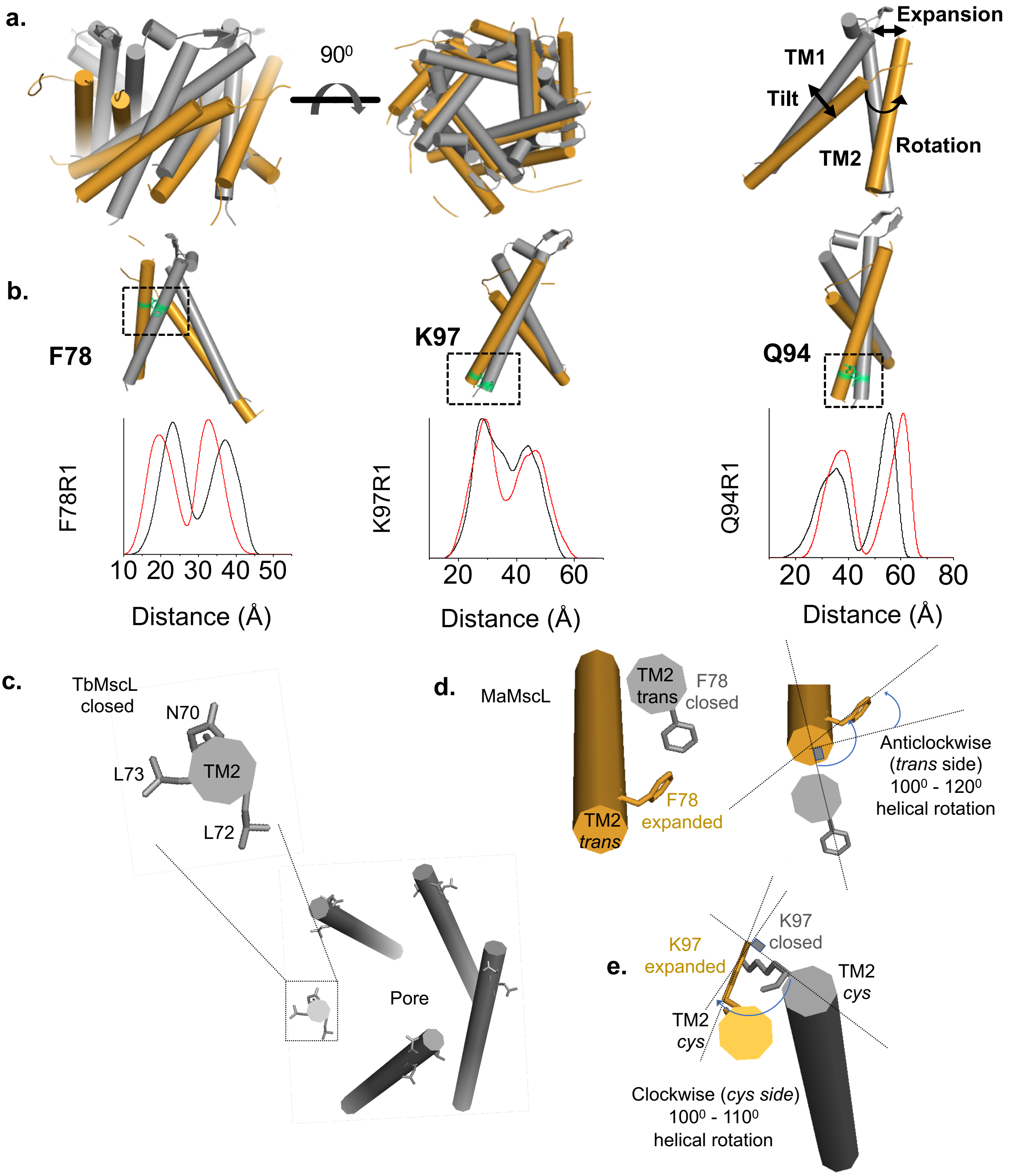

Supplementary Figure 7: MaMscL and TbMsc structural transition similarities a. Side (left) and pore (middle) view of MaMscL closed (PDB 4Y7K, grey cylinders) and expanded (PDB 4Y7J, golden cylinders) structures superimposed. Single monomers (right) are shown for clarity **b.** F78, K97 and Q94 residues shown on MaMscL's TM2 (top, dashed line box) and MTSSLWizard modelled distances of closed (black line) and expanded (red line) MaMscL R1 mutants (bottom). Closed MaMscL-F78 (top) has an orientation similar to that of TbMscL-L72R1 and experiences a similar change (reduction of distances after expansion). Orientation of TbMscL-K100 lies between those of closed MaMscL-K97 and –Q94. Both residues show little changes in their modelled distance distributions after expansion, consistent with PELDOR measurements on TbMscL-K100R1/L89W, as shown in Figure 4a. **c.** TbMscL TM2 periplasmic side pore view and orientation of N70, L72 and L73 (upper TM2) residues combined with L89W in our PELDOR studies. **d.** MaMscL F78 equivalent to TbMscL L72 e.g. orientation and location (upper TM2). An anticlockwise TM2 rotation of ~110⁰ is occurring during transition from the closed (outward - lipid facing F78) to the expanded (inward - pore facing F78) state. **e.** Similar TM2 rotation (d) leads to minor or no distance changes for MaMscL K97 (equivalent TbMscL K100). Despite large conformational changes (a) K97 is localised in a similar spot in space for the expanded and closed states. This is also evident in (b), where resulting unchanged modelled distances (MaMscL K97) are shown.

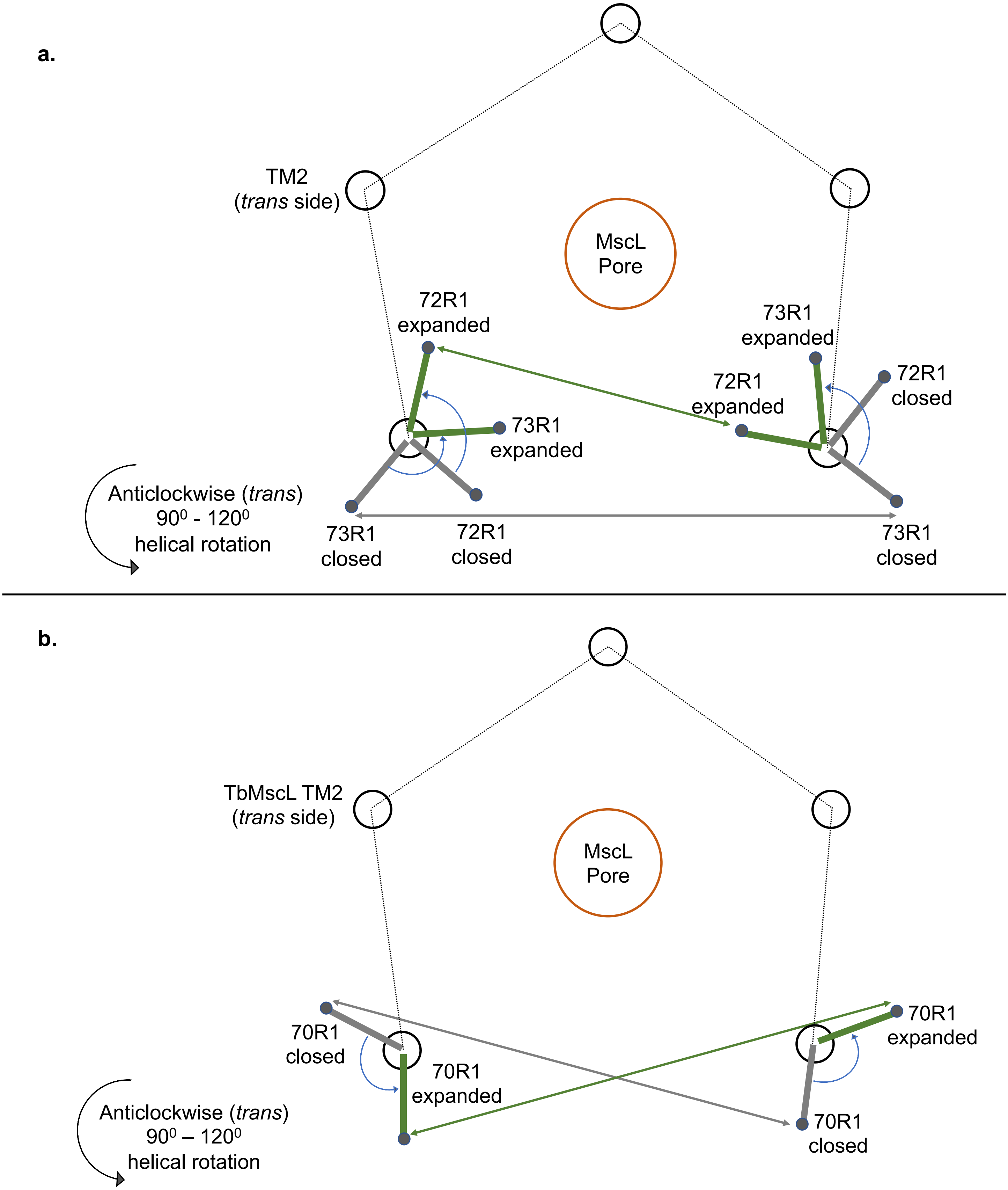

Supplementary Figure 8: Upper TM2 rotation schematic for combined to L89W mutation sites used in our PELDOR studies. a. L72R1 and L73R1 showed a large reduction in distances upon TbMscL opening consistent with a TM2 anticlockwise rotation of ~ 100-120^0, leading to both of them facing inside the pore and resulting in very short distances <20Å. This causes dipolar line broadening in low temperature cwEPR (Fig 4a&b) and is consistent with MaMscL x-ray structures of the closed and expanded states (Fig S8). **b.** N70R1 showed no significant distance changes again consistent with a upper TM2 rotation of ~110-120^0 , taking into account its initial side chain orientation (Fig S8c) in the TbMscL x-ray structure (PDB 2OAR), leading to both closed and expanded orientations facing away from the pore, towards the lipid bilayer.

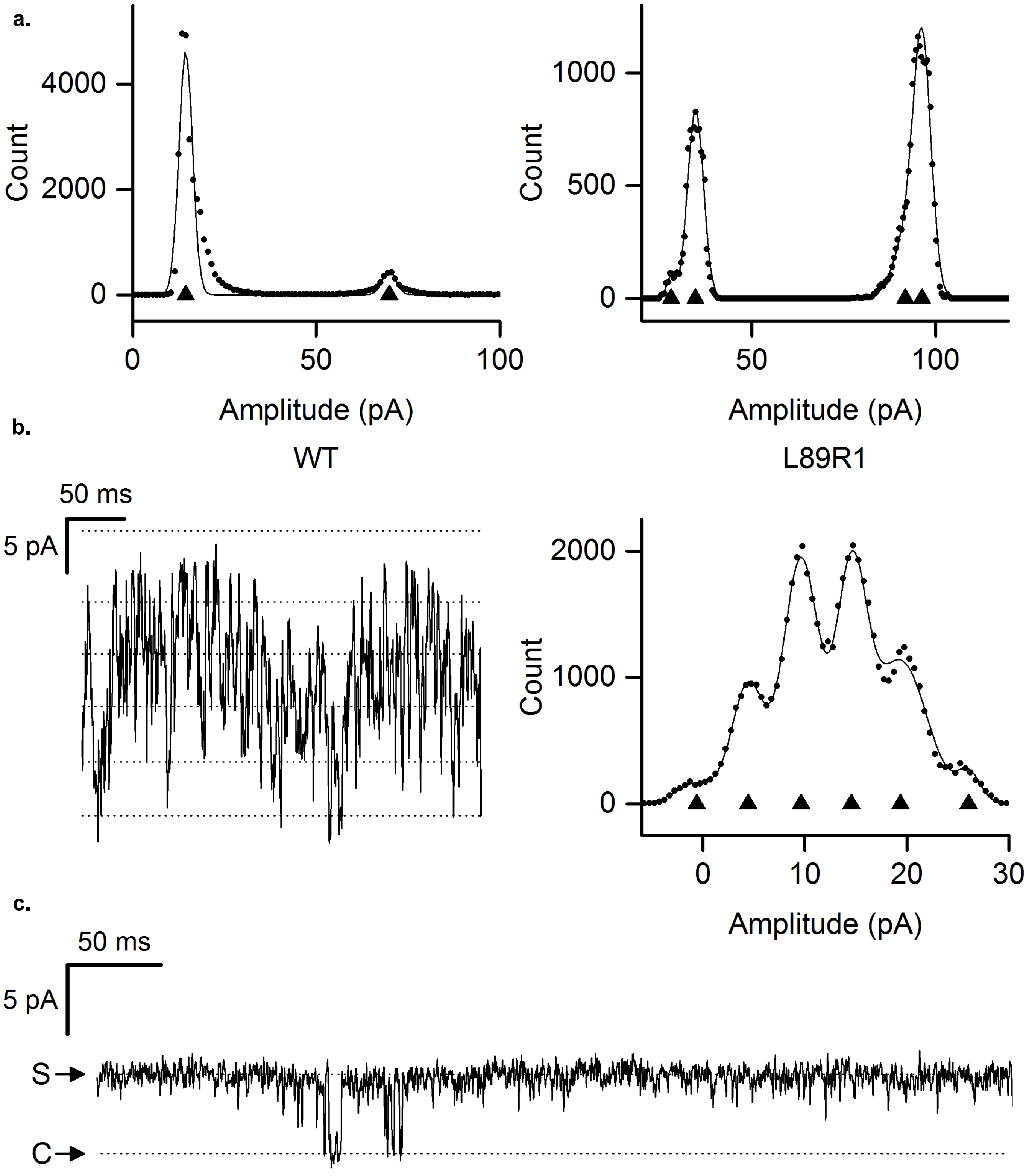

Supplementary Figure 9: Single molecule patch clamp recordings and analysis. a. Exemplar amplitude histograms from recordings at 20 mV from wild-type and L89R1 TbMscL, as indicated, and fitted with the sum of Gaussian distributions (*solid line*), with the means of the individual peaks indicated (*upright triangles*). The inter-peak differences representing the channel full openings are 55 pA and 62 pA for WT and L89R1, respectively. With L89R1, the full openings are superimposed on a constitutively-active sub-conductance channel current of approximately 5 pA. **b.** Representative constitutively-active channel currents recorded at 20 mV with no pressure applied to the patch pipette. The dashed lines represent the means of the Gaussian distributions (*solid line*) fitted to the amplitude histogram (*upright triangles*). The mean inter-peak difference is 5.3 ± 0.34 pA (n=5). **c.** Representative recording at +20 mV from a patch excised from GUV containing L89W TbMscL, with no externally-applied pressure. The brief downward deflections (C), approximately 5 pA, are closures from an otherwise constitutively active sub-conductance state (S).

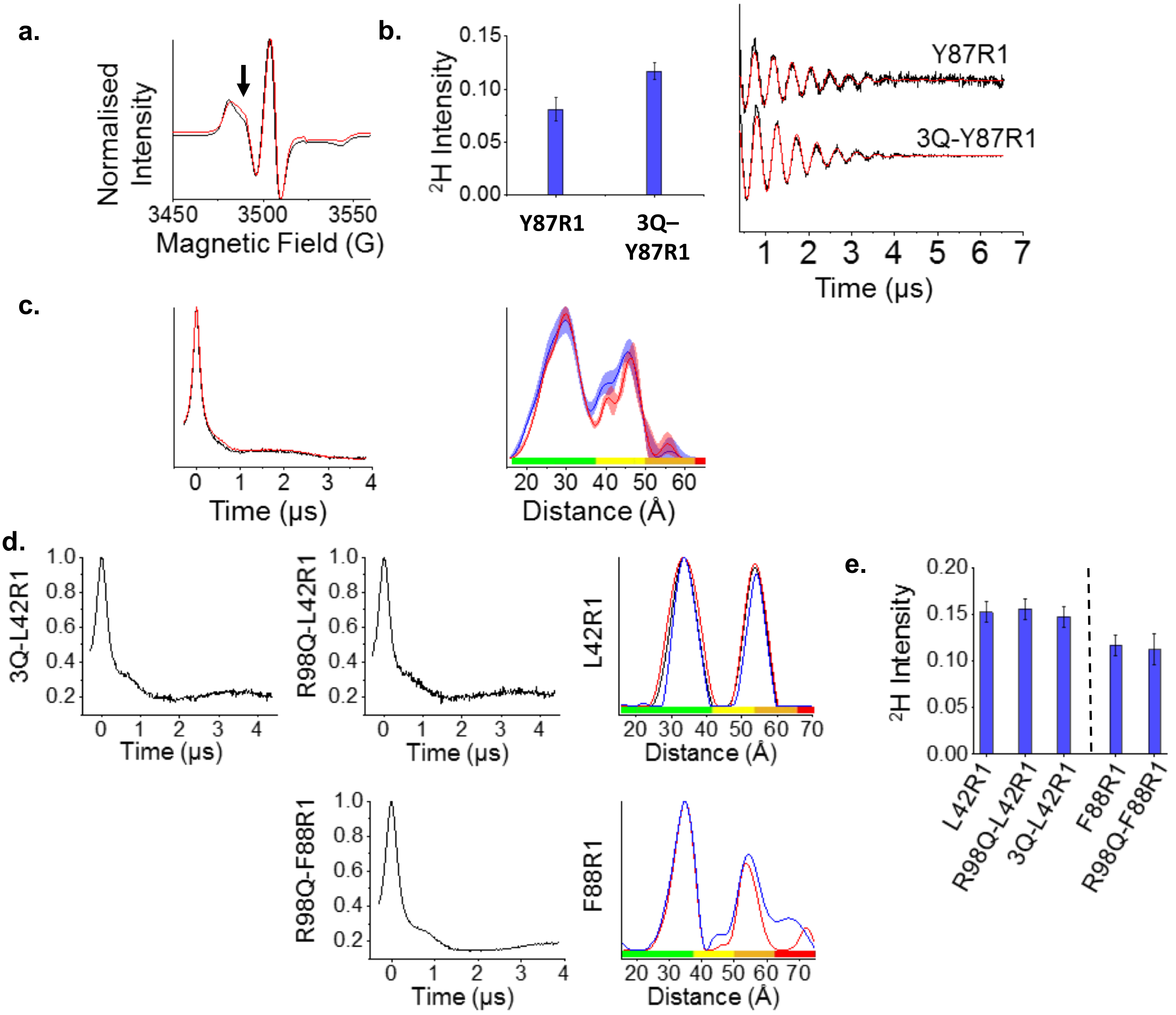

Supplementary Figure 10: The effect of lipid headgroup binding on MscL conformation a. cwEPR spectra of Y87R1 (black) and 3Q-Y87R1 (red). The black arrow shows the increase in mobility of the 3Q-Y87R1 spectrum. **b.** 3pESEEM ^{2}H accessibility of the multiple mutants compared to single-cysteine ones **c.** Left: Comparison of uncorrected raw time-domain PELDOR traces of Y87R1 (black) and 3Q-Y87R1 (red). Right: Distance distributions comparison (Y87R1 in blue, 3Q-Y87R1 in red). **d.** Time-domain traces of R98/K99/K100Q-L42R1 (left) and R98Q-L42R1 and R98Q-F88R1 (middle). On the right, distance distributions of the single R1 mutants (blue lines) are compared to those of the equivalent 3Q (black line) and/or R98Q (red lines) paired mutant. **e.** 3-pulse ESEEM ^{2}H signal intensity comparison between Q, 3Q and single cysteine mutants. Errors are based on the residual of the fitted model between the experimental time – domain traces and the damped harmonic oscillation function used for fitting.

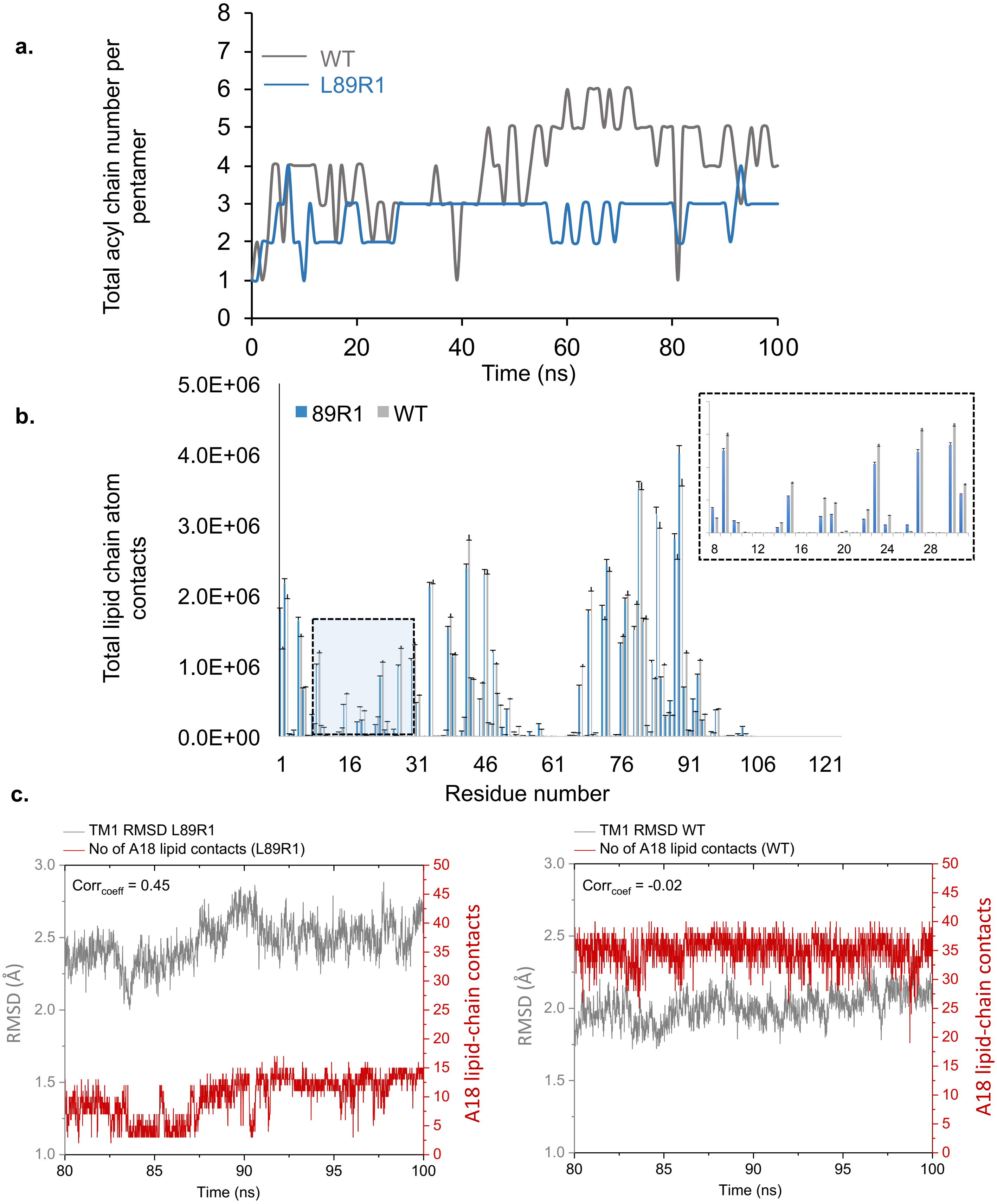

Supplementary Figure 11: Total lipid acyl-chain contact comparison between WT and L89R1 TbMscL. a. Comparison of the number of DDM single acyl chains residing within the NPs over time (100 ns atomistic MD simulation), between WT (grey) and L89R1 (blue) line TbMscL channels. **b.** Total number of lipid-chain atom contacts over the 100 ns MD simulations per TbMscL residue for L89R1 (blue) and WT (grey). NP forming residue region is highlighted in transparent light blue square and magnified (inset). **c.** Correlation between RMSD (Å) of the TbMscL pore-lining helix states (grey line) and lipid-chain contacts with A18 (NP-lipid chain occupancy, red line), over the last 20 ns of the MD simulation.

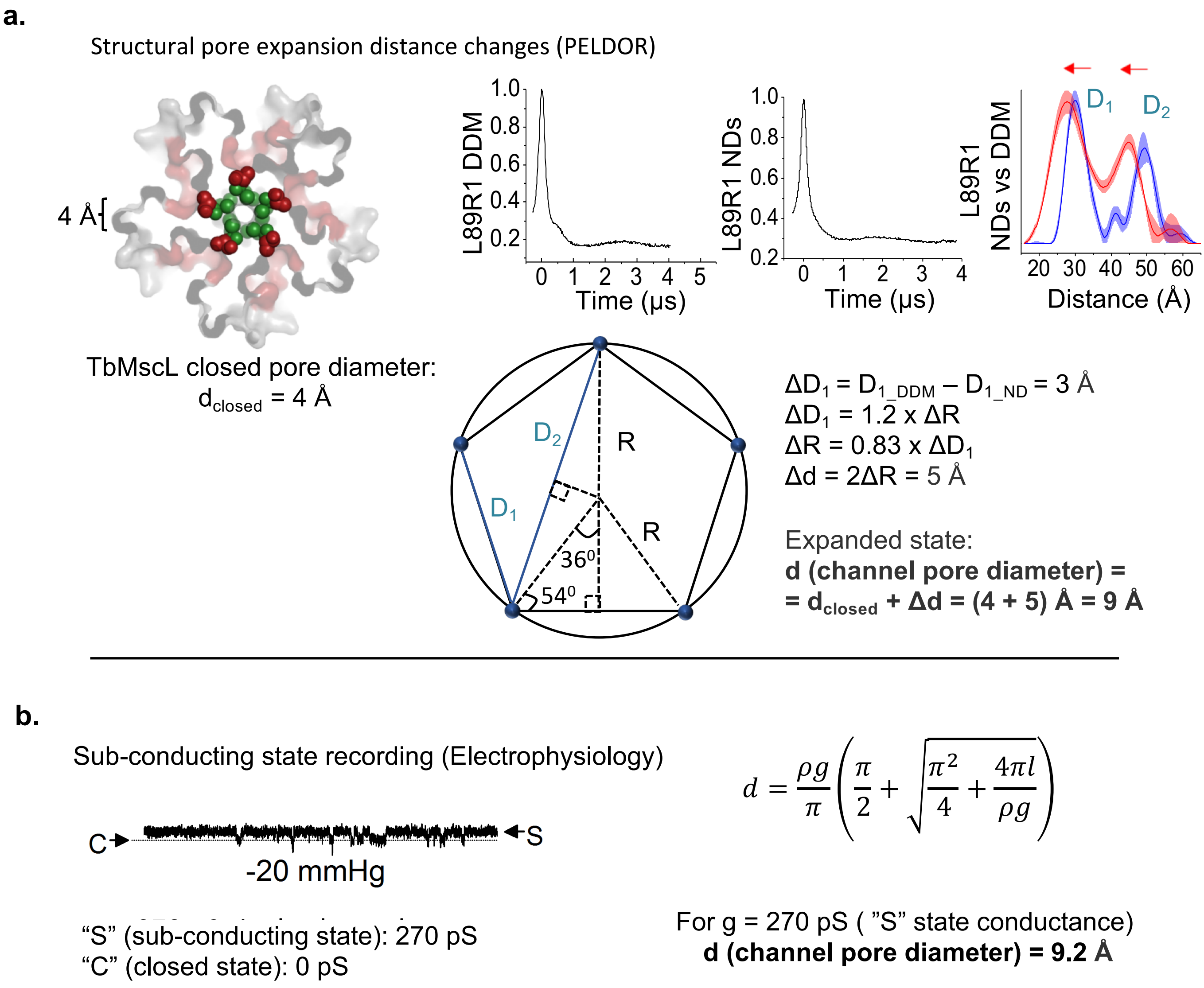

Supplementary Figure 12. Pore dimensions agreement between the expanded PELDOR state and the single-channel sub-conducting state. a. structural state transition (closed to expanded) observed by PELDOR for L89R1 in the absence (DDM, blue line) *vs* presence of bilayer compression (nanodiscs, red line) and **b.** sub-conducting state recorded by electrophysiology at low or baseline (lipid-glass adhesion) tension (Fig 5a & S9). Calculations for the total pore diameter based on PELDOR distance measurements (a) for L89R1 (D_1) between DDM (absence) and nanodiscs (presence) of bilayer compression, using formulas for pentameric C5 channel symmetry[1]. Pore diameter of the closed state TbMscL (PDB 2OAR)[2], x-ray model for L17 (green spheres) is ~4 Å. Pore diameter for the "S" state (b) was performed using the substate "S" measured conductance (electrophysiology, L89R1) of 270 pS and using parameter values for MscL[3]. Similar values (i.e. 8.8 Å) were obtained for L89W "S" substate (250 pS), observed at baseline tensions and pore dimensions derived from D_2 distance measurements. These values are in agreement with the pore dimensions of the expanded MaMscL x-ray structure (PDB 4Y7J)[4]. PELDOR time-domain traces and distributions are adopted from Fig. 2 and 3.

Supplementary Tables

TbMscL modified mutant	Labeling efficiency (%)
L2R1	3
F5R1	15
F34R1	3
F79R1	6
A85R1	8
Y87R1	79
F88R1	67
L89R1	62
E102R1	81

Supplementary Table 1 : Spin labelling efficiency (MTSSL) of representative TbMscL mutants

TbMscL modified mutant	D_1 (Å)	D_2 (Å)	Ratio (D_2/D_1)
L42R1	33.5	54.5	1.62
L73R1	32.1	52.2	1.63
Y87R1	29.7	45.4	1.53
F88R1	34.6	54.5	1.57
L89R1	30.0	49.3	1.64
R98R1	21.7	32.6	1.50
K99R1	32.9	51.3	1.56
K100R1	34.2	51.7	1.51
E102R1	29.2	46.7	1.60
V112R1	19.6	30.6	1.56

Supplementary Table 2: D_2/D_1 distance ratio of modified TbMscL mutants (Theoretical value is 1.6 for C5 versus 1.4 for C4 and 1.7 for C6)

Mutant	D	f	Dc	fc
L2R1	0.198	2	0.027	3%
F5R1	0.31	15	0.251	19%
F34R1	0.108	3	0.055	6%
F79R1	0.095	6	0.107	5%
A85R1	0.074	8	0.141	4%
Y87R1	0.715	79	0.839	58%
F88R1	0.84	67	0.775	79%
L89R1	0.796	62	0.743	71%
E102R1	0.766	81	0.849	66%

Supplementary Table 3: Experimental modulation depths (Δ) and labelling efficiencies (f) for selected mutants. Fit of a single modulation depth parameter (λ = 0.46) and assuming a pentamer (n = 5, see methods) leads to the back-calculated modulation depths parameter (Δ_c) and labelling degree (f_c) based on f and Δ, respectively. Comparison between experimental and calculated values indicates decent agreement and consistency with a pentameric structure.

TbMscL modified mutant	PA 29:0/:1	PG 30:1	PG 32:0/:1	Extra
WT	✓		✓	PE 30:0/:1
L2R1		✓	✓	
F5R1	✓	✓	✓	
L23R1	✓	✓	✓	
F34R1			✓	
L42R1	✓	✓		
V48R1		✓	✓	
L72R1	✓	✓	✓	
F79R1	✓	✓		
F84R1	✓	✓	✓	PE 29:0
A85R1		✓		
Y87R1	✓	✓		PG 24:1
F88R1	✓	✓	✓	
L89R1		✓		
L89R1		✓	✓	PG 33:1<br>PG 34:1<br>PG 37:2
R98R1		✓		
K100R1				PE 33:1<br>PG 36:2
E102R1		✓		PG 33:1
V112R1		✓	✓	

Supplementary Table 4 : ES-MS lipid analysis of purified TbMscL samples. Phosphatidic acid (PA) was detected along with PG and PE lipids. Number of acyl chain carbon atoms and double bonds is noted next to each lipid.

		Lennard Jones / Non-bonded Energy (KJ/mol)	Coulomb Energy (KJ/mol)	Total Energy (KJ/mol)	Total Energy Ratio (WT/L89R1)	Lennard Jones Error (KJ/mol)	Coulomb Error (KJ/mol)	Total Energy Error (KJ/mol)
**NPs (A18) & lipid chain**	WT TbMscL	-20.0448	2.01458	-18.03022	**109.62**	0.53	0.091	0.621
	L89R1 TbMscL	-0.345626	0.181141	-0.164485		0.052	0.014	0.066
**L89 & lipid chain**	WT TbMscL	-182.822	-21.333	-204.155	**0.64**	1.8	3.3	5.1
	L89R1 TbMscL	-299.306	-19.4744	-318.7804		2.9	0.93	3.83
**NPs (A18) & L89**	WT TbMscL	-0.103436	0.0230408	-0.0803952	**0.36**	0.041	0.017	0.058
	L89R1 TbMscL	-0.189256	-0.0309416	-0.2201976		0.018	0.003	0.021
**L89 & TM1**	WT TbMscL	-16.8117	3.73575	-13.07595	**0.18**	0.51	0.08	0.59
	L89R1 TbMscL	-74.775	4.31228	-70.46272		1.6	0.41	2.01

Supplementary Table 5: Pairwise energy calculations between a) NP and lipid, b) L89 and lipid and c) NP and L89 d) L89 and TM1 based on the 80-100ns of the fully atomistic molecular dynamic simulations

TbMscL mutant	Primers (5’ - 3’)
L2C	ACTTTTAGGAGGTAAAAACCATGTGC
F5C	CCATGTTGAAAGGCTGTA AAGAATTCCTGGC
I23C	CGGTAATATTGTTGACTTGGCGGTTGCG
F34C	GCTGGTGACGAAGTGCACCGATTCCATCATCACG
L42C	CCATCATCACGCCGTGCATCAATCGTATCG
V48C	CAATCGTATCGGCTGCAACGCGCAAAGC
N70C	CGATTTGTGTGTCCTGCTGAGCGC
L72C	CGATTTGAATGTCTGCCTGAGCGCCGCTA
L73C	GTCCTGTGCAGCGCCGCTATTAACTTCTTTCTGATCGC
F79C	GCGCCGCTATTAACTGCTTTCTGATCGC
F84C	CTTTCTGATCGCATGTGCCGTCTATTTCCTG
A85C	CTGATCGCATTTTGCGTCTATTTCCTG
Y87C	ACTTCTTTCTGATCGCATTTGCCGTCTGC
F88C	GCTATTAACTTCTTTCTGATCGCATTTGCCGTCTATTGC
L89C	GTCTATTTCTGCGTTGTGCTGCCGTACAACACCC
R98C	CGTACAACACCCTGTGCAAAAAGGGTGAGG
K99C	GTACAACACCCTGCGCTGCAAGGGTGAGGTC
K100C	CACCCTGCGCAAATGCGGTGAGGTCGAACAG
E102C	CGCAAAAAGGGTTGCGTCGAACAGCCAGGT
V112C	CGAACAGCCAGGTGACACGCAAGTGTGC
R98Q	CGTACAACACCCTGCAAAAAAAGGGTGAGGTC
R98Q/K99Q/K100Q	CGTACAACACCCTGCAACAACAGGGTGAGGTCG
L89W	CTATTTCTGGGTTGTGCTGCCGTACAACACC

Supplementary Table 6: Primer sequences used for TbMscL site directed mutagenesis (SDM) in this study

Supplementary References

1. Pliotas, C. Ion Channel Conformation and Oligomerization Assessment by Site-Directed Spin Labeling and Pulsed-EPR. *Methods Enzymol* **594**, 203-242, doi:10.1016/bs.mie.2017.05.013 (2017).

2. Chang, G., Spencer, R. H., Lee, A. T., Barclay, M. T. & Rees, D. C. Structure of the MscL homolog from Mycobacterium tuberculosis: a gated mechanosensitive ion channel. *Science* **282**, 2220-2226 (1998).

3. Cruickshank, C. C., Minchin, R. F., Le Dain, A. C. & Martinac, B. Estimation of the pore size of the large-conductance mechanosensitive ion channel of Escherichia coli. *Biophys J* **73**, 1925-1931, doi:10.1016/s0006-3495(97)78223-7 (1997).

4. Li, J. *et al.* Mechanical coupling of the multiple structural elements of the large-conductance mechanosensitive channel during expansion. *Proc Natl Acad Sci U S A* **112**, 10726-10731, doi:10.1073/pnas.1503202112 (2015).
