# Supplementary material for: Allosteric activation of an ion channel triggered by modification of mechanosensitive nano-pockets
Source: Nat Commun. 2019 Oct 10;10:4619. doi: 10.1038/s41467-019-12591-x (PMC6787021; doi:10.1038/s41467-019-12591-x)
Supplement: Supplementary file 5 — Description of Additional Supplementary Files [file 41467_2019_12591_MOESM5_ESM.pdf]

Title: Supplementary Movie 1:
Description: Lipid chain penetration into the NPs for WT TbMscL channel. Top pore view (periplasmic side) of representative atomistic molecular dynamic simulations between the 90–100 ns of the simulation for WT TbMscL. DMPC lipids are depicted in cyan sticks and protein (MscL) which is taken into account in the simulations, is not illustrated for clarity in observing the effect L89 (non-modified/native Leu, WT) has, on the NP-penetrating inner-leaflet lipid chains. For the majority of the simulation lipid chains point towards the pore axis and their access to the NPs remains unrestricted.

Title: Supplementary Movie 2:
Description: The effect of NP-entrance modification (L89R1) to inner-leaflet lipid chains. Top pore view (periplasmic side) of representative atomistic molecular dynamic simulations between the 90–100 ns of the simulation for L89R1 TbMscL. DMPC lipids are depicted in cyan sticks and protein (MscL) which is taken into account in the simulations, is not illustrated for clarity in observing the effect L89R1 modification has, on the NP-penetrating inner-leaflet lipid chains. Most of the latter bent away from the channel pore axis in presence of the L89R1 modification, in contrast to WT MscL simulation (Supplementary Movie 1), during which lipid chains point towards the pore axis and their access to the NPs remains unrestricted.
